# Overexpression of PCNA Attenuates Oxidative Stress-Caused Delay of Gap-Filling during Repair of UV-Induced DNA Damage

**DOI:** 10.1155/2017/8154646

**Published:** 2017-01-01

**Authors:** Yi-Chih Tsai, Yi-Hsiang Wang, Yin-Chang Liu

**Affiliations:** Institute of Molecular Medicine, National Tsing Hua University, Hsinchu 30013, Taiwan

## Abstract

UVC irradiation-caused DNA lesions are repaired in mammalian cells solely by nucleotide excision repair (NER), which consists of sequential events including initial damage recognition, dual incision of damage site, gap-filling, and ligation. We have previously shown that gap-filling during the repair of UV-induced DNA lesions may be delayed by a subsequent treatment of oxidants or prooxidants such as hydrogen peroxide, flavonoids, and colcemid. We considered the delay as a result of competition for limiting protein/enzyme factor(s) during repair synthesis between NER and base excision repair (BER) induced by the oxidative chemicals. In this report, using colcemid as oxidative stress inducer, we showed that colcemid-caused delay of gap-filling during the repair of UV-induced DNA lesions was attenuated by overexpression of PCNA but not ligase-I. PCNA knockdown, as expected, delayed the gap-filling of NER but also impaired the repair of oxidative DNA damage. Fen-1 knockdown, however, did not affect the repair of oxidative DNA damage, suggesting repair of oxidative DNA damage is not of long patch BER. Furthermore, overexpression of XRCC1 delayed the gap-filling, and presumably increase of XRCC1 pulls PCNA away from gap-filling of NER for BER, consistent with our hypothesis that delay of gap-filling of NER attributes the competition between NER and BER.

## 1. Introduction

UVC irradiation or numerous carcinogenic chemicals-caused DNA adducts are repaired by nucleotide excision repair (NER). NER consists of a cascade of events including initial damage recognition, dual incision to excise the damage containing oligonucleotide, gap-filling, and ligation [1, 2]. We have previously shown that oxidants such as hydrogen peroxide, menadione, and other chemicals including colcemid, amoxicillin, and flavonoids of propolis can inhibit gap-filling during the repair of UVC-induced DNA lesions [3]. For such study, the gap-filling was blocked by DNA synthesis inhibitors hydroxyurea and Ara-C [4, 5]. Such blockage results in the accumulation of repair intermediates with the gap, which can be detected by methods such as single-cell gel electrophoresis, also called the comet assay [6, 7]. If the DNA synthesis inhibitors are removed, the gap-filling will be quickly restored. Our previous studies indicate that the restoration of gap-filling can be delayed by oxidants or chemicals which have capacity to produce oxidative DNA damage [3, 8]. That type of damage can be detected by the comet assay with incubation of formamidopyrimidine glycosylase (Fpg) and endonuclease III (Endo III), which are bacterial enzymes that recognize oxidized purines and pyrimidines, respectively [9]. We have linked the repair of oxidative DNA damage, that is, base excision repair (BER), with the delay of gap-filling [3]. The delay is absent in BER deficient cells, for example, EM9 (in which XRCC1 is defective), and is restored if XRCC1 is supplemented. Moreover, while the gap-filling of NER is delayed by BER, repair of oxidative DNA adducts is not slowed down by NER, suggesting that the delay is probably not because of shortage of nucleotide precursors (dNTPs); if so, both NER and BER would have been affected. Rather, for some reason BER is dominant over NER.

Many types of DNA adducts including oxidative lesions are repaired by BER [2, 10]. Like NER, BER consists of sequential steps including damage recognition and removal by glycosylase, strand cleave by AP lyase or AP endonuclease, 3′ or 5′ ends polishing, and gap-filling by either short patch (for 1 nucleotide) or long patch repair (for 2–15 nucleotides) [11, 12]. For short patch repair, the gap is filled by DNA polymerase *β* assisted by XRCC1 and Lig-III. For long patch repair, the gap is filled by DNA polymerase *ε*/*δ* assisted with PCNA, Fen-1, and (ligase-1) Lig-I. As the two excision repair mechanisms potentially share common machinery during gap-filling, we proposed that the delay is a consequence of competition for the limiting molecules (illustrated in Figure S1 in Supplementary Material available online at https://doi.org/10.1155/2017/8154646). PCNA, known as the sliding clamp of DNA polymerases [13], is involved in many aspects of DNA metabolism [14]. PCNA is essential to NER during gap-filling. Although involvement of PCNA in BER appears quite limited, numerous studies have indicated that PCNA interacts with almost all the players of BER including glycosylases, AP endonuclease, and XRCC1 [15–17].

Therefore, we consider PCNA the candidate of the limiting molecules linked with the delay. Besides, Lig-I and Fen-1 may be the limiting factors if repair of oxidative DNA damage involves long patch pathway of BER. Results of our study indicate that overexpression of PCNA attenuates the delay. Similar effect was not found with overexpression of Lig-I. Furthermore, knockdown of Fen-1 did not impair the repair of oxidative stress-induced DNA damage. Thus, PCNA but not Lig-I or Fen-1 is required in repairing both UV and oxidative stress-caused DNA damage and becomes factors limiting for NER and BER, when these two kinds of DNA damage are induced at the same time.

Induction of both NER and BER simultaneously increased cell death compared to that of NER or BER alone. Overexpression of PCNA could reduce the cell death. Since overexpression of PCNA is prevalent in malignant tumors [18], our study provides insight from view of DNA repair that PCNA may be used as target of chemotherapy.

## 2. Materials and Methods

### 2.1. Cell Cultures, Expression Plasmids, and Antibodies and UV Irradiation

Human gastric adenocarcinoma AGS cells or human lung adenocarcinoma CL 1-0 cells were cultured in the conditions as those described previously [3]. Both cell lines were originally obtained from the American Type Culture Collection (Manassas, VA, USA). These two cell lines were used because of the availability and also the consideration of transfection: CL 1-0 cells appeared easier for obtaining stable transfectant compared to AGS cells. The expression plasmid of PCNA, pEGFP-N3-PCNA, was constructed with rat PCNA cDNA [19] at the* BamH*I and* EcoR*I sites of the expression vector pEGFP-N3.

pEGFP-N3-ΔPCNA for PCNA without the C-terminal 39 amino acids was constructed from rat PCNA cDNA with polymerase chain reaction using the primers: forward GGGCGGCGTGAACCTACAG; reverse GCTCCCCACTCGCAGAAAACT. Lig-1 expression plasmid in pc1079-pmRFP was obtained from H. Leonhardt (Ludwig Maximilians University). To knock down expression of PCNA or Fen-1 the respective plasmids to produce short hairpin RNA of PCNA or Fen-1 in pLKO.1 were obtained from National RNAi Core Facility (Genomic Research Center, Taipei, Taiwan). Antibodies to monitor the protein levels of proteins including PCNA, Lig-1, and Fen-1 were provided by Santa Cruz Co. or GeneTex Co. Antibody of 8-oxo-dG was provided by Trevigen Co. (Gaithersburg, MD, USA). Cells were irradiated with a germicidal lamp (Sankyo Denki, Japan, 254 nm). The fluorescence rate of the lamp was 50 mW/cm^2^ calibrated with a UVX-254 radiometer (UVP Co., San Gabriel, CA, USA).

### 2.2. Chemicals

Most of chemicals including hydroxyurea, Ara-C, hydrogen, and peroxide were obtained from Sigma-Aldrich Co., while colcemid was obtained from Invitrogen Co. Most of the chemicals were dissolved in water before use. For studying the effect on gap-filling, cells at 60% confluence were UV irradiated (10 J/m^2^) and were then treated with 2.5 mM hydroxyurea and 25 *μ*M Ara-C (H/A) for 2 h. Colcemid at 50 ng/mL would be used in this study for transient induction of oxidative DNA damage.

### 2.3. Comet Assay

Conventional comet assays (single-cell gel electrophoresis) were performed as described previously [20] with modifications [21]. Preparation of the cell-containing gel (on microscopic slides) and the subsequent cell lysis were carried out as described previously [21]. After cell lysis, the slides were washed three times with deionized water and were denatured in 0.3 N NaOH and 1 mM EDTA for 20 min. Electrophoresis was carried out in the same denaturation solution at 25 V, 300 mA for 25 min. Each slide was rinsed briefly in water, blotted, and then transferred to 0.4 M Tris-HCl, pH 7.5. DNA was stained by adding 20 *μ*L propidium iodide (50 *μ*g/mL) onto the slide. A coverslip was then applied, and the slide was examined using a Fluorescence Microscope (Axioplan 2, Zeiss Co.). Images of at least 50 cells per slide were recorded with a closed-circuit display camera (CoolSNAP). The migration of DNA from the nucleus of each cell was measured with a computer program (http://tritekcorp.com) and is expressed as % DNA in the tail. Data are presented as averages of at least three independent experiments ± standard error.

### 2.4. Comet Assay with Enzymes

To measure oxidative DNA adducts with the alkaline comet assay, we performed an additional step immediately after the cell lysis step, that is, incubating the slides containing the nucleoids with Endo III and Fpg (from Trevigen Co.; 2 units of each enzyme per slide in buffer with 10 mM Tris-HCl pH 7.4) as described previously [9]. Endo III and Fpg are bacterial glycosylases that specifically recognize oxidized pyrimidines and purines, respectively.

### 2.5. Flow Cytometric Analysis of Nucleoid Size

This method is based on the phenomenon that nucleoid size becomes larger when cellular genome has damage. The original protocol [22] was used with modification. Immediately following lysis each sample was stained with propidium iodide (PI; 20 *μ*g/mL) and was analyzed in a Becton-Dickinson FACS 440 flow cytometer connected to an Apple microcomputer using peak height analog to digital conversion. Nucleoids were passed through a 594 nm argon laser beam at up to 150–300 nucleoids/s, and triggering on fluorescence, forward scatter, side scatter, and total PI fluorescence were collected. Some fluorescence nucleoid histograms were obtained using a Becton-Dickinson FACScan with analog to digital conversion carried out by area integration rather than peak height. The information is presented in the form of a frequency histogram.

### 2.6. ELISA of 8-OHdG

The method described previously [23] was followed with some modifications. In brief, cells were treated with 1 mM H_2_O_2_ for 1 h and harvested at the indicated time points for genomic DNA extraction. Each DNA sample was denatured at 95°C for 5 min and was then chilled on ice followed by incubation with 2 units of alkaline phosphatase (BioLabs, Ipswich, MA, USA) and 5 units of DNase I (Sigma-Aldrich) in 50 mM Tris, pH 7.3, and 1 mM MgCl_2_ (Merck, Germany) buffer at 37°C for 2 h. 96-well plates were first coated with 0.003% protamine sulfate (Sigma-Aldrich) and then with 100 ng 8-OHdG (Sigma-Aldrich). Coated wells were added in a series of concentrations of pure 8-OHdG or DNA samples. The antibody to 8-OHdG (1 : 500; Trevigen), biotin goat anti-mouse IgG (1 : 1000; Zymed Laboratories, Inc., San Francisco, CA, USA), and peroxidase-streptavidin (1 : 10000; Sigma-Aldrich) were used sequentially for the detection of 8-OHdG. O-Phenylenediamine (Pierce, Thermo Scientific, Rockford, IL, USA) dissolved in citrate phosphate buffer (5.103 g citrate acid monohydrate, 7.297 g Na_2_PO_4_ in 1 L ddH_2_O adjusted to pH 5.0 with citric acid) was used as a substrate for peroxidase. The absorbance was read at 492 nm with a microplate reader (Molecular Devices, Sunnyvale, CA, USA).

### 2.7. Immunostaining of 8-OHdG

The method described previously [24] was followed with some modifications. In brief, cells seeded for 1 day were treated with 1 mM H_2_O_2_ for 1 h and were harvested at the indicated time points for immunostaining. Cells after fixation were treated with 4 N HCl to denature DNA. The antibody to 8-OHdG (1 : 500) and a secondary antibody conjugated with Hilyte Flour 488 (1 : 200; Ana Spec Inc., Fremont, CA, USA) were used for detecting 8-OHdG. Nuclei were counterstained with 4′,6-diamidino-2-phenylindole (Sigma-Aldrich). Fluorescence images were captured by a digital camera on a Fluorescence Microscope (Zeiss/Axioskop 2 Mot plus).

### 2.8. Flow Cytometry Analysis of Cellular DNA Contents and the Measurement of BrdU Incorporation

The experiment was done based on the procedures described previously [25] In brief, human AGS cells were cultured with 5-bromo-2′-deoxyuridine (BrdU) (Sigma, USA) for 3 h. After being washed with phosphate buffer saline (PBS), the cells were stored in 70% EtOH at −20°C overnight. Then, the cells were incubated in RNAse A (Sigma, USA) at 37°C 30 min to remove RNA. For denaturing DNA, the cells were treated with HCl-Triton (0.1 N HCl + 0.7% triton X-100) solution 10 min at ice and subsequently heated at 97°C in sterile water 2 min and put on ice for 15 min. The cells were then incubated with 1 : 20 anti-BrdU (GTX, number 28039) solution at 37°C for 30 min, then, reincubated with 1 : 20 100 mL secondary antibody, mouse-FITC (GTX, number 85337), and washed with PBST (PBS + 5% FBS + 0.5% Tween 20). For DNA staining, the cells were resuspended in propidium iodide (PI) containing solution. The cell cycle profile and the BrdU incorporation were analyzed simultaneously by flow cytometry (FACS Calibur, Becton-Dickinson, Franklin Lakes, NJ).

## 3. Results and Discussion

**Figure 1:**
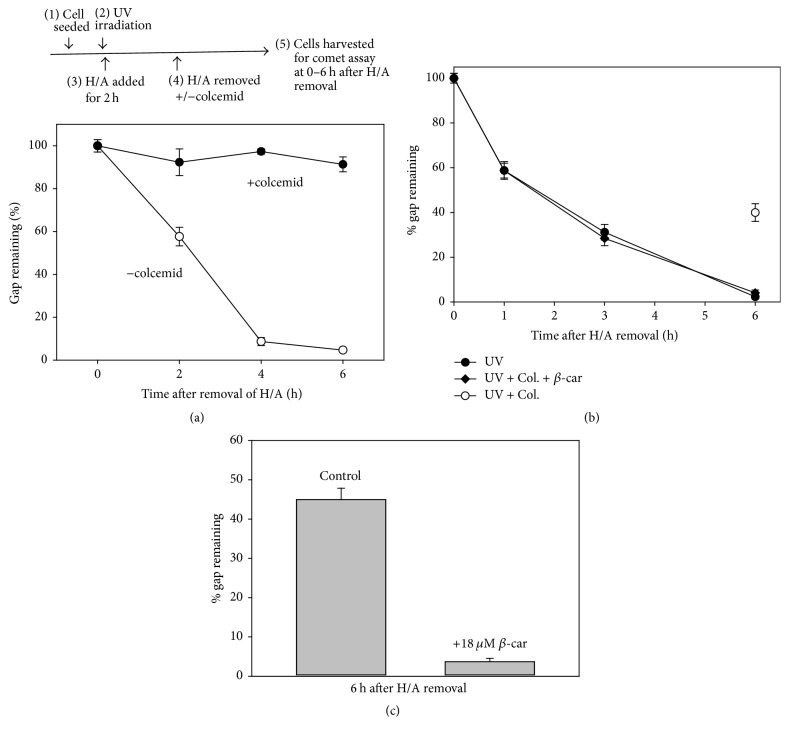
(a) Top: schematic illustration of experimental protocol for studying the effect of chemicals on gap-filling during repair of UV-induced DNA lesions. Bottom: colcemid delays the gap-filling. Initial level of the gaps at the time zero after H/A removal was taken as 100%. (b) Similar to (a), AGS cells in log phase were UV irradiated (3 J/m^2^) and H/A treated for 2 h. After H/A was removed, cells were treated with or without colcemid or 18 *μ*M *β*-carotene. Cells were harvested for comet assay at the indicated time. (c) Similar to (b), cells were harvested at 6 h after H/A was removed for comet assay.

Initially while studying the activity of tumor suppressor p53 in Chinese hamster ovary cell line K1, we noticed in serendipity that mitotic inhibitor, colcemid, caused a moderate increase of UV-induced cell death [26]. Further study indicates that colcemid though does not affect the excision of DNA adducts, it delays the gap-filling as shown in Figure 1. The intermediates of gap-filling were accumulated by the presence of hydroxyurea and Ara-C (H/A). Removal of H/A resulted in quick decrease of % gap remaining (the open circles, Figure 1(a)). The % gap remaining was calculated from the ratio between the level of DNA breaks detected at a certain time following H/A removal and the level of DNA breaks detected immediately before removal of H/A. Most of gaps remained unfilled in the presence of colcemid even at 6 h after H/A was removed (the closed circles, Figure 1(a)). Moreover, we found that not only colcemid but also amoxicillin and flavonoids of propolis showed similar inhibitory effect upon gap-filling. The commonality of these chemicals is their capacity to cause oxidative DNA damage (see Figure S2). Addition of antioxidant such as *β*-carotene abolished the colcemid-caused delay of gap-filling (Figure 1(b)): the rates of gap-filling between the treatment of UV alone and the treatment of UV plus colcemid and *β*-carotene were indistinguishable (Figure 1(b); compare the curve of closed circles and the curve of closed diamond). Also, as shown in Figure 1(c), while >40% of gaps remained unfilled in the control experiment, most of the gaps were filled when *β*-carotene was added.

Thus, since the antioxidant was able to mitigate the effect of colcemid in gap-filling, the effect of colcemid in gap-filling is more likely attributed to its prooxidant capacity rather than its effect on mitotic spindles.

### 3.1. Overexpression of PCNA but Not Ligase-1 Attenuates the Delay of Gap-Filling

**Figure 2:**
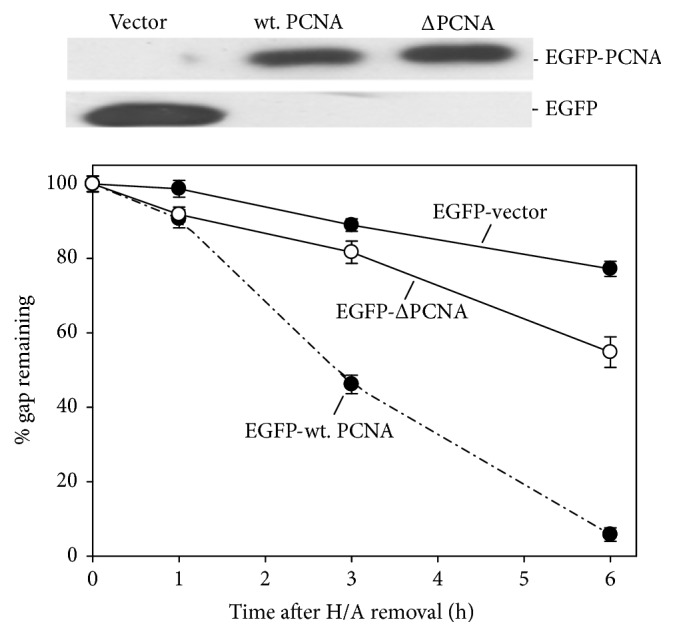
Top: western analysis of PCNA protein levels in total cell extracts of the CL1-0 cells stably transfected with wild type PCNA (EGFP-wt. PCNA) or truncated PCNA (EGFP-ΔPCNA) expression plasmids or EGFP-vector alone. Bottom: overexpression of PCNA attenuates colcemid-caused delay of gap-filling. CL1-0 cells stably transfected with the indicated plasmids were treated with the procedures illustrated in Figure 1 for studying the effect of PCNA overexpression on colcemid-caused delay of gap-filling during repair of UV-induced DNA lesions.

**Figure 3:**
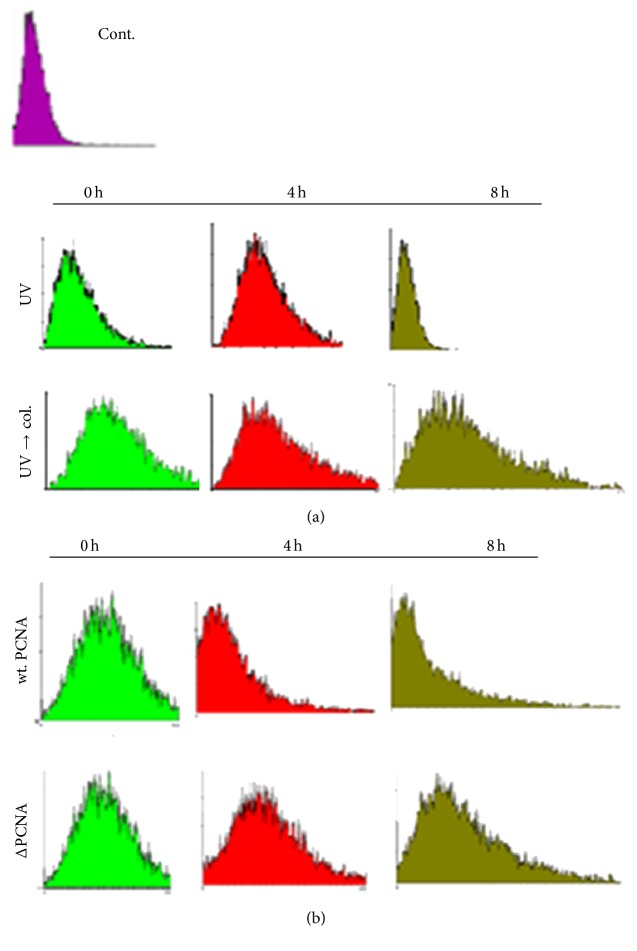
Flow cytometric analysis of nucleoid size. AGS cells were treated similarly as those described in Figure 1. (a) Cont. for control, that is, cells without treatment. UV and UV → col. for UV alone and UV then colcemid, respectively. Time points: 0–8 h after H/A removal. (b) Similar to (a), yet the cells were transiently transfected with the indicated expression plasmids. (Histogram: *x*-axis: forward scattering; *y*-axis: counts.)

**Figure 4:**
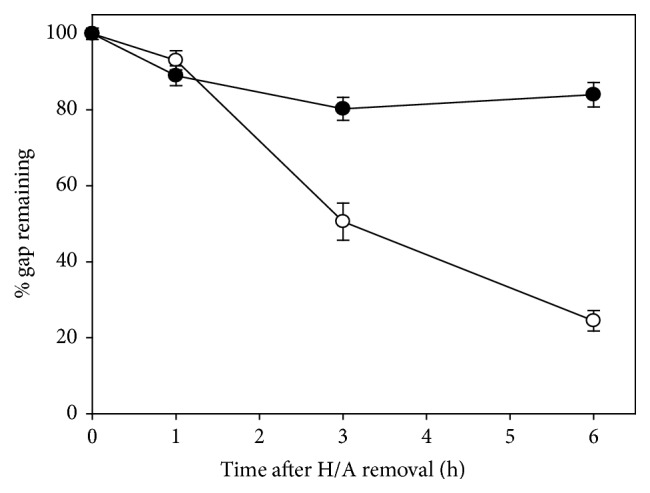
The effect of PCNA knockdown on gap-filling during repair of UV-induced DNA lesions. Similar to the procedures described in Figure 1, yet colcemid was excluded to avoid “overdelay.” AGS cells were stably transfected with pLKO-1 shPCNA to knock down PCNA (closed circles) or control vector (open circles).

**Figure 5:**
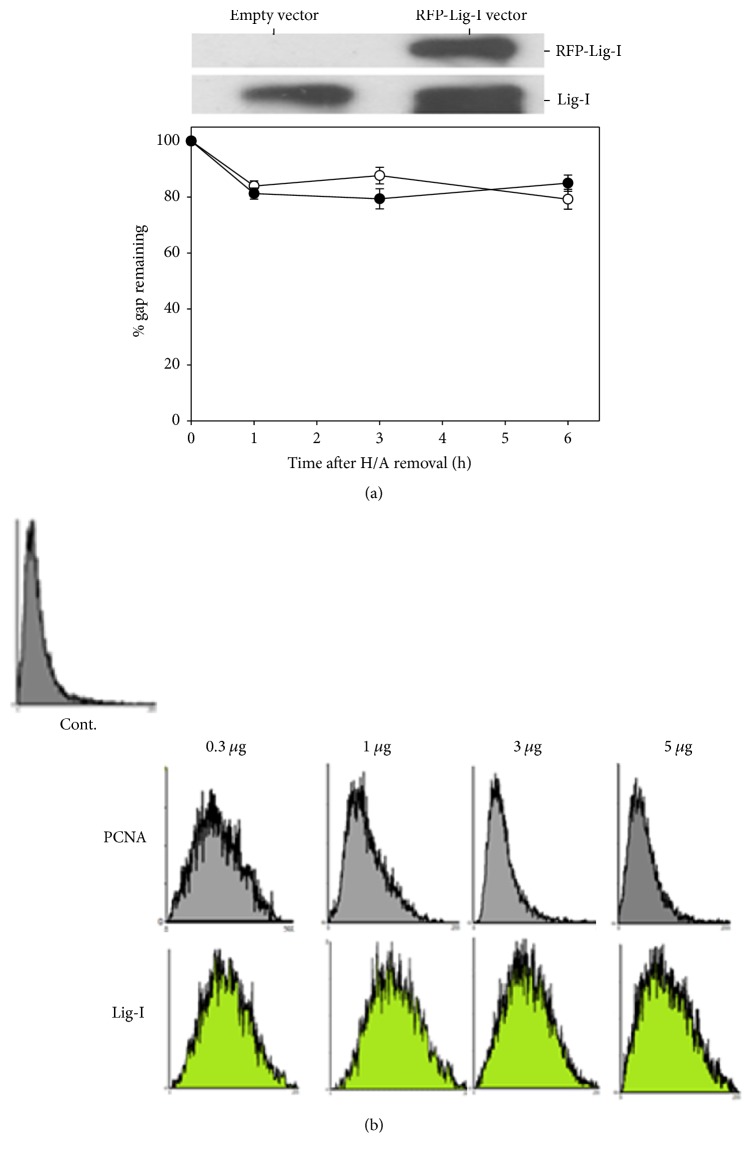
The effect of Lig-1 overexpression on colcemid-caused delay of gap-filling during repair of UV-induced DNA lesions. (a) Top: western analysis of Lig-1 protein in CL1-0 cells stably transfected with expression plasmid of Lig-1 (RFP-Lig-1) or empty vector. Bottom: similar to Figure 1, yet the CL1-0 cells stably transfected with expression plasmid of Lig-1 (filled circle) or empty vector (open circle) were used. (b) Flow cytometric analysis of nucleoid size. Dose dependence. Cells were transiently transfected with various amounts of PCNA or ligase-I expression plasmids.

To know if PCNA is a limiting factor of gap-filling during NER in the presence of BER, we tested if overexpression of PCNA would attenuate the delay of gap-filling. For the study, the expression of PCNA in nuclei was examined by the immunostaining (Figure S3). Human CL 1-0 cells stably transfected with PCNA expressing or control plasmids were treated with UV irradiation, H/A, and colcemid as described previously. Overexpression of PCNA in the form of EGFP fusion protein markedly reduced the delay of gap-filling, whereas EGFP alone had no similar effect (Figure 2). As compared to wild type PCNA, truncated form of PCNA (ΔPCNA, with deletion of C-terminal 39 amino acids) showed much less effectiveness to attenuate the delay. C-terminal region of PCNA has been shown to be involved in interaction with ligase-1 or Fen-1 [27–29]. The attenuation effect of PCNA on the delay of gap-filling was also detected with the flow cytometry-based nucleoid size analysis. DNA strand breaks relax chromatin structure leading to increase of nucleoid size. If UV irradiation is done alone, nucleoid size is restored to control pattern at 8 h after H/A removal; presence of colcemid delayed the restoration (Figure 3(a)). Expression of wild type PCNA but not ΔPCNA restored the nucleoid size to nearly the control pattern at 8 h after H/A removal and in the presence of colcemid (Figure 3(b)). PCNA knockdown, in contrast, greatly delayed the gap-filling during repair of UV-induced DNA damage (Figure 4), encapsulating the necessity of PCNA to gap-filling of NER. Overexpression of ligase-I (Lig-1), however, failed to produce attenuation effect in comet assay (Figure 5(a)) and also in nucleoid size analysis regardless of the increase of Lig-1 expression vector (Figure 5(b)). Thus, Lig-1 may not be a limiting factor of gap-filling during NER in the presence of BER.

### 3.2. PCNA Is Essential to Repair of Oxidative Stress-Induced DNA Lesions

**Figure 6:**
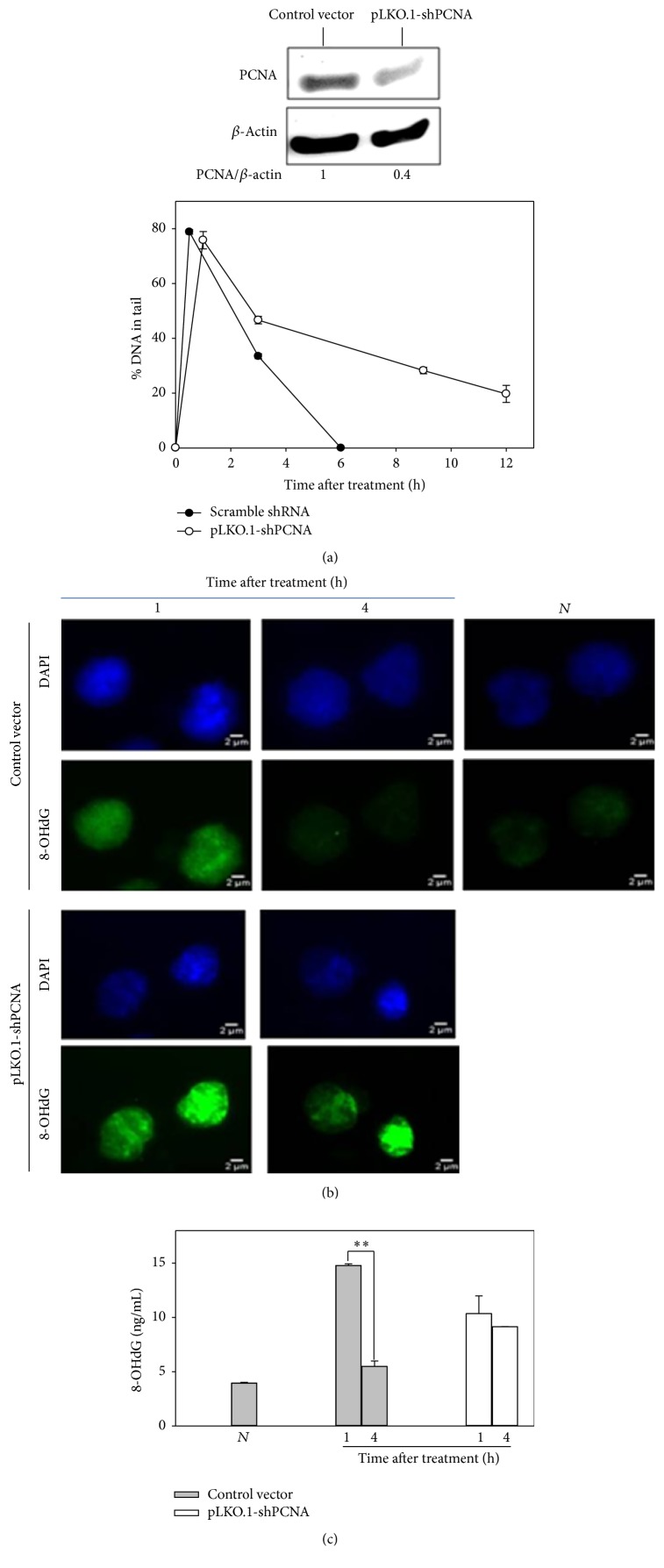
The effect of PCNA knockdown on the repair of oxidative DNA damage. (a) Comet assay. AGS cells transfected with pLKO-1 shPCNA to knock down PCNA or control vector were treated with 20 *μ*M H_2_O_2_ for indicated periods of time before being harvested for comet-Fpg/Endo III assay to monitor the levels of H_2_O_2_-induced DNA lesions in cells. Bottom panel: western analysis of PCNA protein with levels of *β*-actin protein as a loading control. (b) Immunostaining analysis and (c) ELISA. Cells transfected with pLKO-1 shPCNA or control vector were treated with 1 mM H_2_O_2_ for 1 and 4 h before being harvested for immunofluorescence and ELISA assays of 8-oxo-dG. Typical images were shown. *N* for control, that is, cells without treatment. *∗∗* for *p* < 0.005.

The above results indicate that PCNA is critical to gap-filling during repair of UV-induced DNA lesions. Increase of the abundance of PCNA facilitated gap-filling presumably because the level of the molecules was sufficient for both NER and BER. Although PCNA has been shown to be involved in long patch pathway of BER, it is unclear whether PCNA is essential for repairing the colcemid-induced oxidative DNA damage. To test if PCNA is necessary for repairing the oxidative stress-induced DNA damage, we examined the effect of PCNA knockdown on repair of Fpg/Endo III sensitive sites in H_2_O_2_-treated cells. PCNA knockdown greatly delayed the repair of oxidative stress-induced DNA lesions (Figure 6(a)). The effect of PCNA knockdown on BER was also confirmed with immunostaining and ELISA methods by monitoring the levels of 8-oxo-dG, a representative oxidized product of bases (Figures 6(b) and 6(c)).

### 3.3. The Repair of Oxidative Stress-Induced DNA Lesions Is Not Fen-1 Dependent

**Figure 7:**
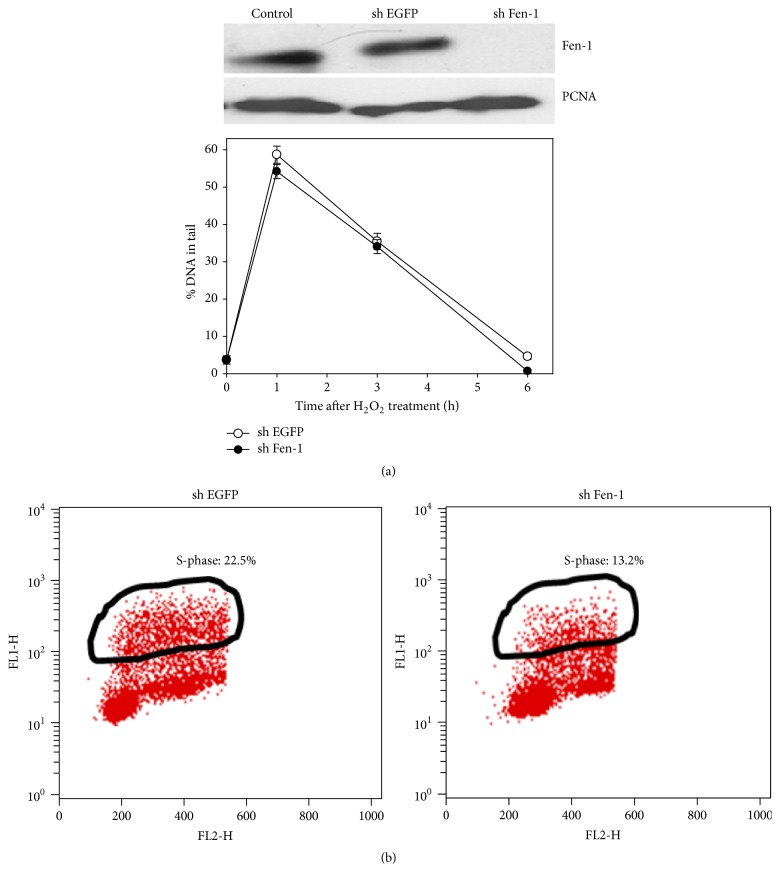
The effect of Fen-1 knockdown. (a) On repair of oxidative DNA damage. Top: western analysis of Fen-1 protein in AGS cells transfected with pLKO-1 sh Fen-1 to knock down Fen-1 or control vector; levels of PCNA protein were used as a loading control. Bottom: comet assay. AGS cells were treated with 20 *μ*M H_2_O_2_ for indicated periods of time before being harvested for comet-Fpg/Endo III assay. (b) On DNA replication. S-phase cells were marked.

In contrast, knockdown of Fen-1, the endonuclease to remove flap structure during DNA replication, did not affect the repair of oxidative stress-induced DNA lesions (Figure 7(a)), though it reduced the fraction of S-phase as measured by BrdU incorporation (Figure 7(b)) as expected. Taking together, our data suggest that repair of oxidative stress-induced DNA damage may not be of long patch repair. It is of interest to know in our future study how PCNA is involved in repair of oxidative stress-induced DNA lesions. Our preliminary data indicate that repair of oxidative stress-induced DNA damage is PCNA and replication dependent (data not shown), consistent with the observation from biochemical study by other investigators that repair of oxidized bases is coupled with replication [16]. We speculate that PCNA may work together with XRCC1 to serve as scaffold for glycosylases/ligase in the situation.

### 3.4. Overexpression of XRCC1 Delayed the Gap-Filling

**Figure 8:**
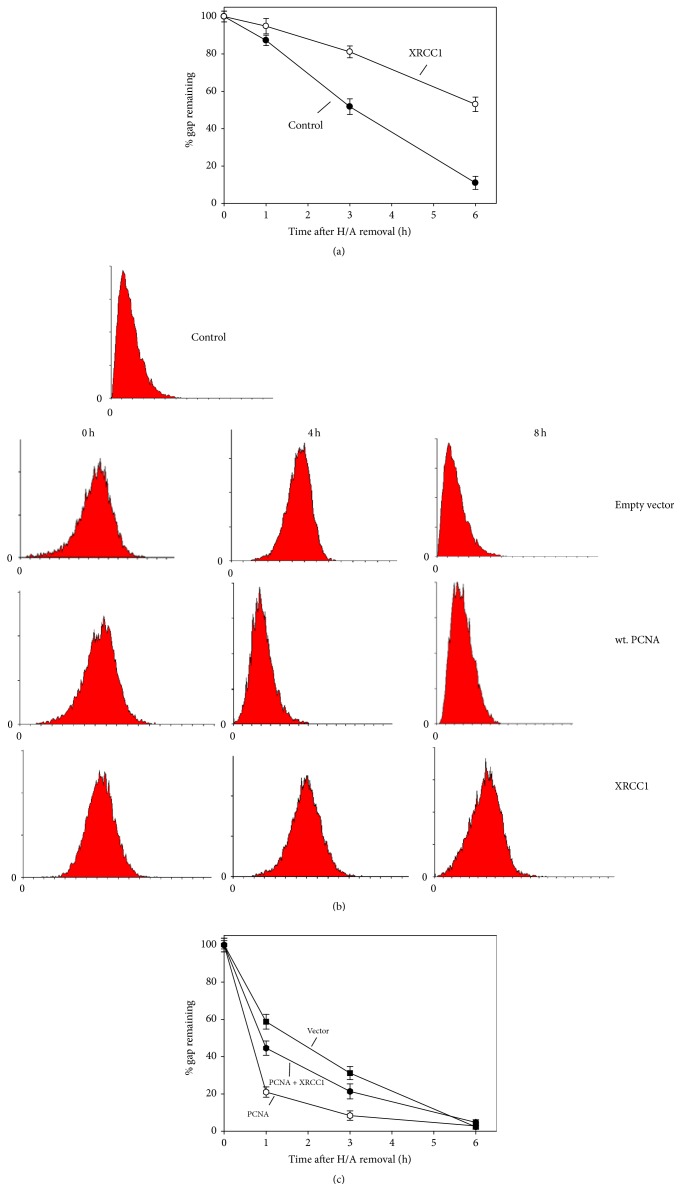
The effect of XRCC1 overexpressed on the gap-filling during repair of UV-induced DNA lesions. ((a) and (b)) Similar to the procedures described in Figures 1 and 3, yet colcemid was excluded. (a) AGS cells were transiently transfected with pCMV XRCC1 plasmids (open circle) or not (closed circle). (b) Cont. for control, that is, cells without treatment. AGS cells were transfected with wt. PCNA or pCMV-XRCC1 or empty vector. Time points: 0–8 h after H/A removal. Histograms of both panels: *x*-axis: forward scattering (relevant to particle size); *y*-axis: cell counts. (c) Similar to procedures described in Figure 1, colcemid was present, and AGS cells transfected with empty vector or plasmid EGF-PCNA with or without pCMV XRCC1 were used.

As mentioned earlier, XRCC1, also known as the scaffold protein of BER, interacts with PCNA [30]. To test whether XRCC1 recruits PCNA for repair of oxidative DNA damage, we examined if overexpression of XRCC1 impaired the gap-filling of NER. The experiment was done as those described in Figure 1: cells were UV irradiated and H/A were added for a period time to prevent gap-filling and then H/A were removed to allow gap-filling to take place. The results indicate that overexpression of XRCC1 delayed the gap-filling. With overexpression of XRCC1, the remaining of gaps was about 60%, while the control had only about 10% at 6 h after removal of H/A (Figure 8(a)). Since the experiment was done with cells without oxidative stress inducer such as colcemid, the result suggests the preferential repair of BER for endogenous level of oxidized bases over NER when XRCC1 was overexpressed. Thus, the level of XRCC1 in ordinary cells must be regulated to avoid the delay of gap-filling of NER. Furthermore, the results (Figure 8(a)) were verified by the flow cytometric analysis of nucleoid size (Figure 8(b)). Consistently, the overexpression of XRCC1 compromised the overexpression of PCNA (Figure 8(c)). The rate of gap-filling in cells transfected with PCNA and XRCC1 was slower than with PCNA alone (Figure 8(c), compare the curve of closed circles and the curve of open circles). Thus, increase of XRCC1 inhibits the gap-filling, and presumably XRCC1 pulls PCNA away from NER for BER. This experiment supports our hypothesis that competition for common components such as PCNA between NER and BER can cause delay of gap-filling of NER.

**Figure 9:**
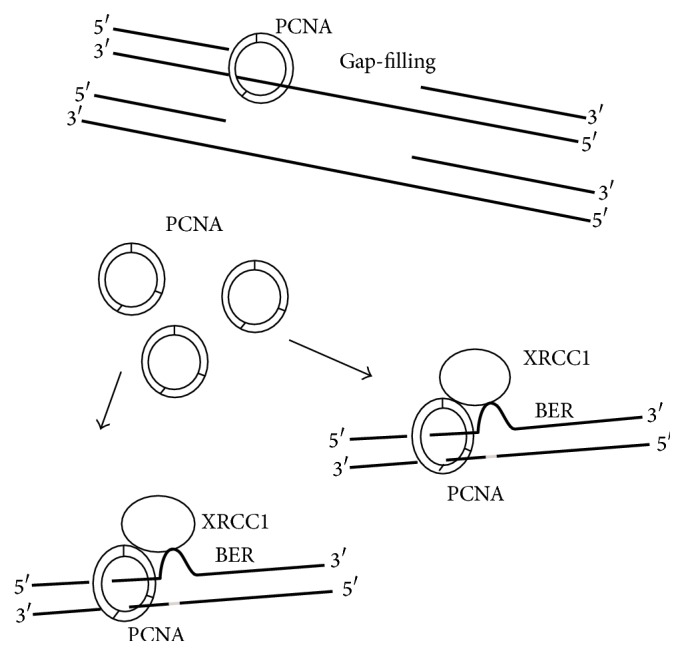
PCNA is important to the gap-filling during the repair of UV-induced DNA injuries and is also essential to the repair of oxidative DNA lesions. When oxidative DNA damage increases or XRCC1 overexpresses, more PCNA is recruited to BER, which may leave more gaps unfilled.

Our observations suggest that PCNA is essential to both NER and BER and becomes limited when excision repairs function. Gap-filling of NER is forced to stand by until BER is completed. Overexpression of PCNA may lift the stringency (Figure 9).

Delay of gap-filling of NER might cause a modest increase of cell death, which could be suppressed by overexpression of PCNA (Supplementary Figure S4). Thus, it is likely that cells may increase expression of PCNA to overcome the trauma. In fact, elevation of PCNA is commonly detected in malignant tumors (e.g., reviewed by [18]). Recently, small molecule inhibitors of PCNA or peptides targeting PCNA have been developed and appear promising for cancer therapy [31, 32].

## Supplementary Material

Fig. S1: Cellar components shared by both NER and BER.
Fig. S2: Immunostaining of PCNA.
Fig. S3: Induction of oxidative stress by colcemid.
Fig. S4: Effect of PCNA on cell viability.
