# Supplementary material for: Overexpression of PCNA Attenuates Oxidative Stress-Caused Delay of Gap-Filling during Repair of UV-Induced DNA Damage
Source: J Nucleic Acids. 2017 Jan 1;2017:8154646. doi: 10.1155/2017/8154646 (PMC5237465; doi:10.1155/2017/8154646)
Supplement: Supplementary file 1 — Fig. S1: Cellar components shared by both NER and BER. Fig. S2: Immunostaining of PCNA. Fig. S3: Induction of oxidative stress by colcemid. Fig. S4: Effect of PCNA on cell viability. [file 8154646.f1.pdf]

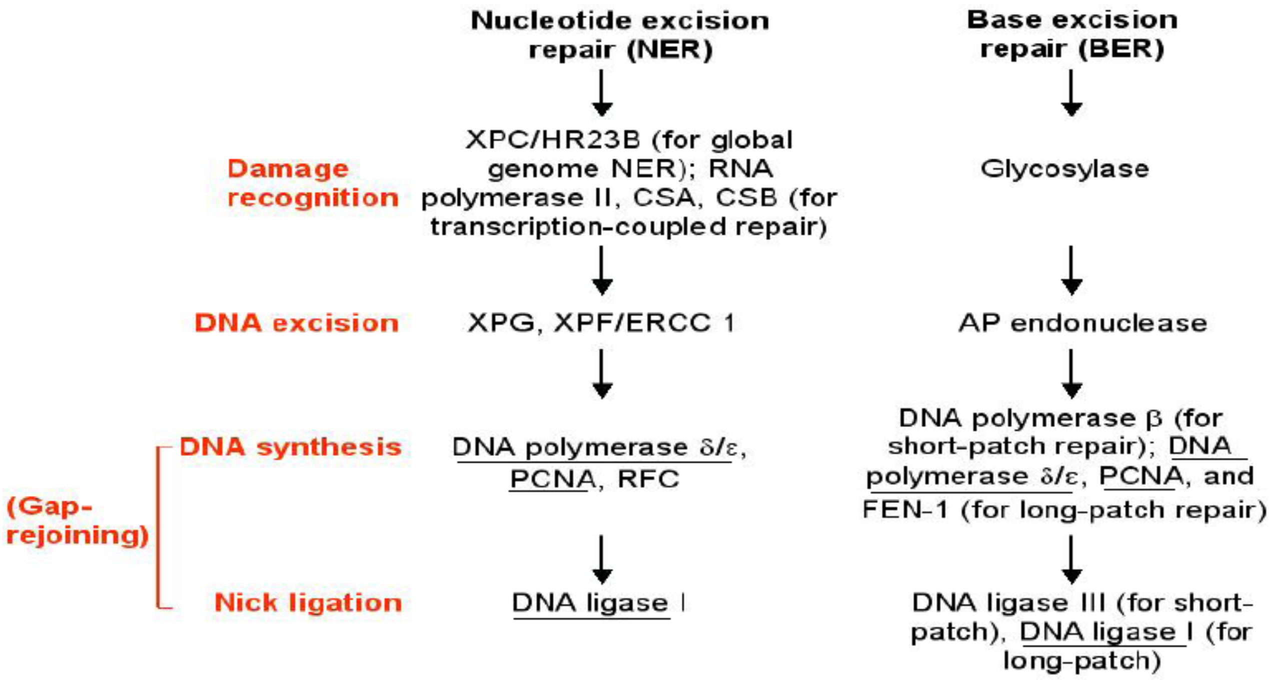

Fig. S1. Cellar components (underlined) shared by both NER and BER.

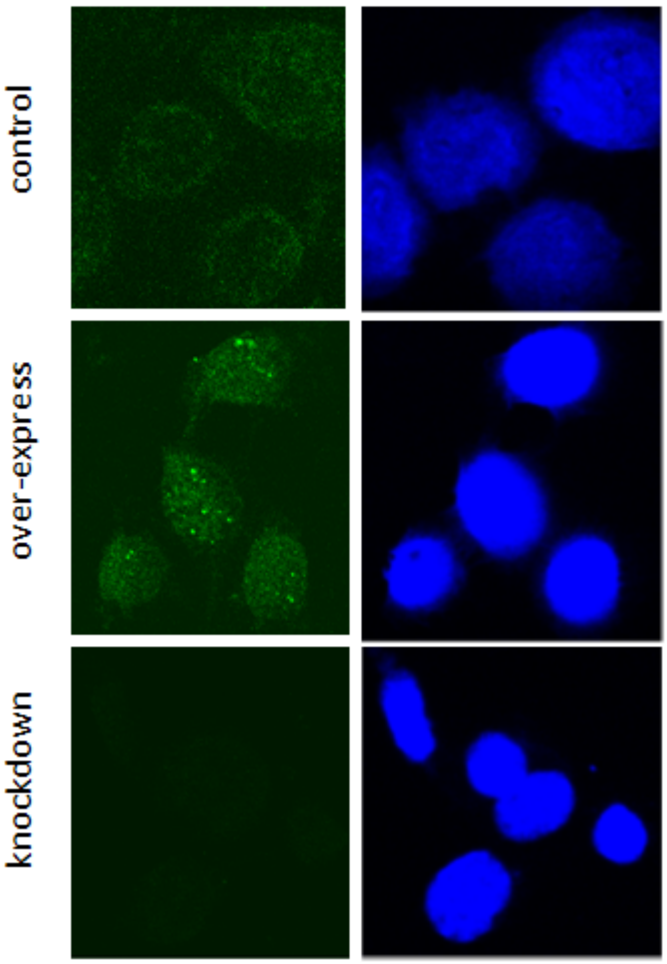

Fig. S2 immunochemical staining of PCNA in cells transfected with empty vector (top panel), wild type PCNA (middle panel) or RNAi PCNA (lowest panel).

A

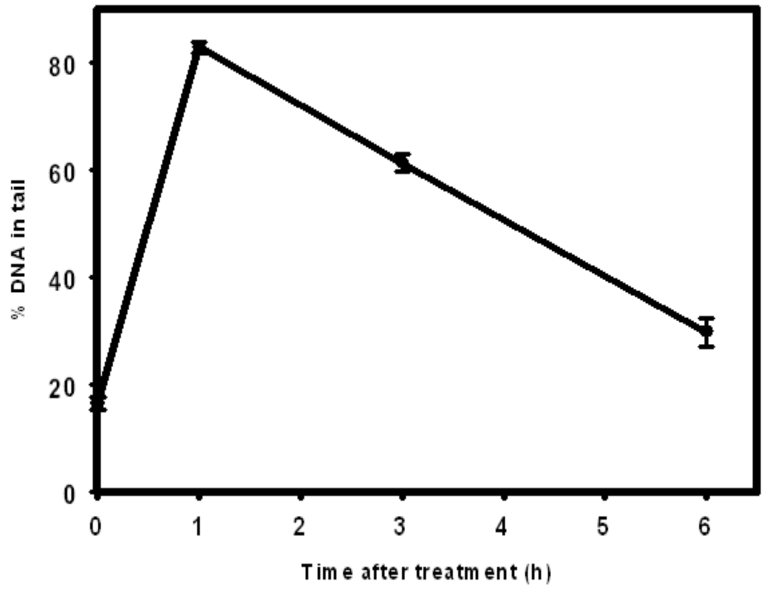

B

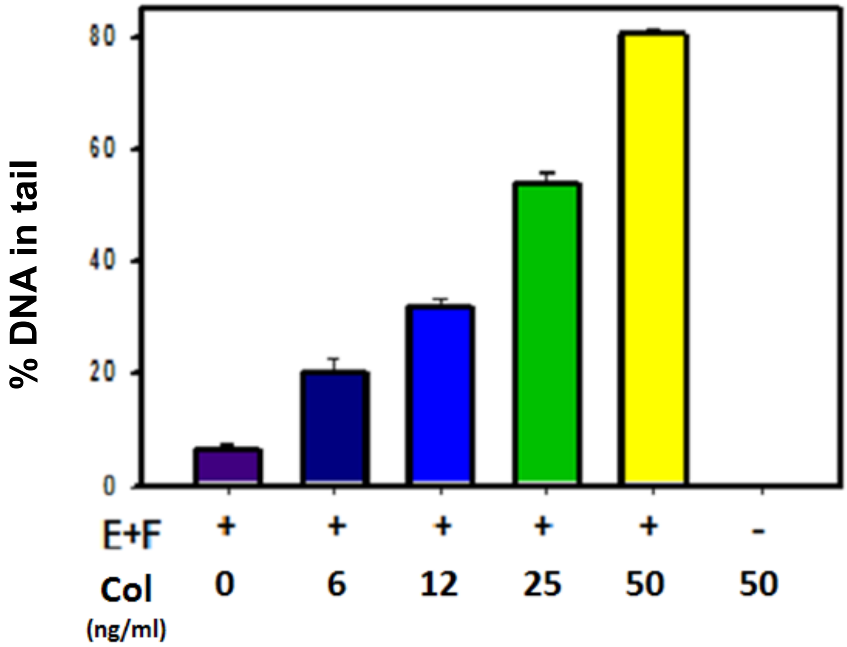

Fig. S3. colcemid-induced oxidative DNA damage (A) kinetics . (B) dose dependence . E+F for endoIII and Fpg Oxidative DNA lesions were determined by comet assay with endoIII and Fpg for specific recognition of oxidized pyrimidines and purines, respectively.

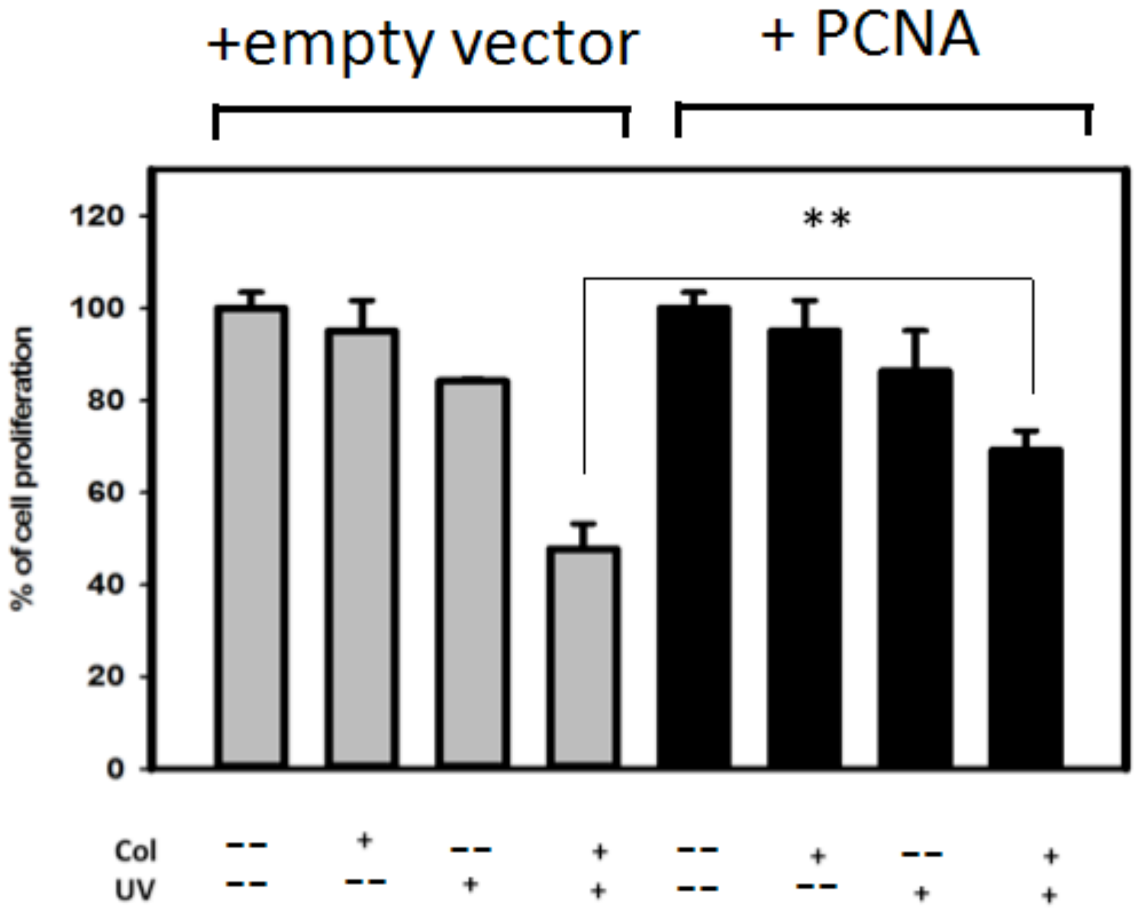

Fig. S4. AGS cells were transfected with EGFP empty vector (gray Bar) or EGFP-wt PCNA (black Bar) were treated with (+) or without (--) colcemid or UV. After 48 h, the cells were harvested for MTS assay. The % cell proliferation is referred to control i.e. no treatment
